# VIM-Polyp: Multimodal Colon Polyp Dataset with Video, Histopathology, and Protein Expression

**DOI:** 10.1038/s41597-025-06168-1

**Published:** 2025-12-03

**Authors:** Refika Sultan Doğan, Ebru Akay, Serkan Doğan, Bülent Yılmaz

**Affiliations:** 1 Department of Bioengineering, Faculty of Life and Natural Sciences, Abdullah Gül University, Kayseri, 38080 Türkiye; 2 Biomedical Instrumentation and Signal Analysis Laboratory, Abdullah Gül University, Kayseri, 38080 Türkiye; 3 Pathology Clinic, Kayseri City Hospital, Kayseri, 38080 Türkiye; 4 Gastroenterology Clinic, Kayseri City Hospital, Kayseri, 38080 Türkiye; 5 GUST Engineering and Applied Innovation Research Center (GEAR), Electrical and Computer Engineering Department, Gulf University for Science and Technology (GUST), Hawally, 32093 Kuwait

**Keywords:** Colon cancer, Diagnostic markers

## Abstract

The dataset in this study includes 202 videos with a total of 422 minutes, reaching Kayseri City Hospital’s gastroenterology department as colonoscopy videos and 1903 microscopy images between 2019 and 2021. It includes 399 colonoscopy, microscopy images, and pathological diagnoses of polyps, as well as immunohistochemical staining results for proteins that play an important role in the assessment of cancerous cells, such as staining results for p53 (clone: bp53-11), Ki-67 (clone: 30-9), CD34 (clone: QBend/10), PD-L1 (clone: SP142), BRAF (clone: V600E) and VEGF (clone: SP125). By sharing the data openly, we aim to facilitate benchmarking, exploratory analysis and transfer-learning studies on colorectal polyps and cancer. In combination with external datasets or pretrained models, the resource can help advance data-driven detection and characterisation work. The diverse range of polyps assigned to cancer stages from 201 patients makes this tool valuable for researchers and clinicians in furthering diagnosis and treatment.

## Background & Summary

Colorectal carcinomas (CRC) represent a common worldwide health problem and rank as the third most common malignancy^1,2^. The pathogenesis in CRC is complex, often involving the formation of a precursor lesion such as colonic adenomas. Accurate detection and characterization of polyps, which are precursors to colorectal cancer, is critical to disease prevention and management^3,4^. These diseases are common digestive tract diseases that demand early detection and diagnosis for emergency medical care with better patient outcomes. Up until now, effective detection has been done through colonoscopy, biopsy, and pathological examination. While these procedures are time-consuming, they are essential due to inter-observer variability. Better diagnostic tools help improve patient care. To address these challenges, digital pathology and machine learning methods have emerged as promising tools^4^. However, there is a lack of publicly available, multimodal datasets combining colonoscopy videos, histopathological images, and molecular markers.

The present study focuses on the analyses of colonoscopy videos and images as sources of information for assessing biopsies related to polyps and cancers. The previous work has established the scope of video-based analysis in the detection and classification of polyps, which provides basic research for developing computer-aided diagnosis systems that leverage video data to enhance accuracy in the detection of polyps^5–7^.

Nowadays, it remains the primary diagnostic tool for the detection, characterization, and follow-up of polyps and cancerous lesions in a completely non-invasive manner. This technique uses a high-resolution video system that captures high-quality, real-time images of the colon’s inner lining. Colonoscopy is an essential tool that provides high-resolution video and image data to evaluate the morphological and structural features of abnormal formations in the colon and rectum^8^. A combination of the datasets with molecular imaging techniques and analytics methods based on AI enables early diagnosis, prognosis assessment, and a determination of the treatment strategy^9,10^. Recent advances in computer vision and machine learning methods have brought hope toward increasing accuracy and efficiency in polyp detection and characterization^7,11^.

Based on this study, it is relevant to identify key literature and former research that have affected the understanding and development of colonoscopy, polyp detection, and cancer diagnosis. However, references^12–14^ form the basis for the importance and novelty of the current study, providing other research and development to follow up in this area. Moreover, the potential usage of the data collected will not be confined to the realm of the current study While the dataset is not large enough for training end-to-end deep learning models, it is well-suited for benchmarking, transfer learning, and cross-modality validation. Table 1. explains publicly available datasets of colonoscopy videos with annotated polyps for polyp detection and classification.

**Table 1:** Publicly available datasets of colonoscopy videos with annotated polyps for polyp detection and classification.

Dataset	Polyp Types	Number of Classes	Publications
CVC-ClinicDB	Yes	3	^52^
CVC-ColonDB	Yes	2	^53,54^
Kvasir	Yes	4	^55^
ETIS-Larib	Yes	5	^56^
ASU-Mayo Clinic	No	-	^57^
CVC-300	Yes	2	^54^
CVC-ClinicDB-2	Yes	4	^58^
KID	Yes	3	^59^
Medico	Yes	2	55
SUN Colonoscopy	Yes	2	^60,61^
REAL-Colon	Yes	3	^62^
PolypsSet	Yes	4	^63^

Accurate diagnosis and classification of colon polyps are crucial in guiding treatment modalities and enhancing patient outcomes. Nonetheless, current datasets commonly show limitations in incorporating multiple modalities, such as colonoscopy videos, histopathology images, and protein expression level results. Protein expression levels were evaluated using immunohistochemistry (IHC). Integrating these structures enhances comprehension of the disease and the cancer-promoting process. Consequently, a multimodal dataset was generated to bridge this gap and assist with research and clinical practice in this field^15^. Previous studies have investigated the histological features of colon polyps and their association with CRC development. However, there are large datasets available that represent high-quality histological images supported by pathological diagnoses. The diagnosis and histopathological classification of colon polyps using biopsy examination is critical in finding treatment and management approaches for these types of growths. However, it is challenging to interpret the images of colon polyp histopathology, given the great expertise in histopathology and good insight into many different polyp types and their characteristics. Besides, somewhat suboptimal inter-observer and intra-observer agreement in interpreting colon polyps can be expected, leading to diagnostic discrepancies. These factors bring out the need for data-driven approaches and computational tools that can assist the pathologist in diagnosing colon polyps by decreasing the workload.

Computer-aided diagnosis systems for abnormality detection in medical images have achieved remarkable success with access to large-labeled datasets in the last decade^15–19^. Supported by the establishment and public releases of numerous datasets, such as mammography, chest X-ray, and ultrasound, machine learning approaches have advanced in many medical applications^20–24^. However, only a few works have focused on creating a dataset of images of colon polyp histopathology, and their numbers are few among the available benchmark datasets.

The first dataset in Table 2, CAMEL^25^, is a weakly supervised learning framework, using only image-level labeled data for histopathology image segmentation. CAMEL uses sample-level label enrichment and a combined multisampling learning approach to address the challenge of obtaining fine-scale labels. The framework consists of two steps: tag enrichment and segmentation. The label enrichment step uses a combined multisampling learning approach to generate a high-quality sample-level dataset. This dataset is used to train a fully supervised classification model. The trained model allows the training of fully managed segmentation models by dividing the original images under the grid and creating labels for the samples. Experimental studies show that CAMEL performs similarly to fully controlled methods on the CAMELYON16 and colorectal adenoma datasets.

**Table 2:** Publicly available datasets of histopathology images with annotated polyps and other tissue types.

Dataset name	Size/content	Modality	Classes/labels
CAMELYON16	177 WSIs	WSI	Metastasis/normal
UniToPatho	9,536 patches	Patches	6 polyp/dysplasia classes
COCAHIS	82 WSIs	Frozen WSI	Tumor/non-tumor
CoNSeP	41 tiles	Tile (1000 × 1000)	Nucleus-level annotations
CRC-TP	22 patients	WSI + patches	Tumor/non-tumor
NCT-CRC-HE-100K	100,000 patches	Patch (FFPE)	9 tissue classes

The second dataset in Table 2 introduces UniToPatho^26^, a comprehensive histopathology dataset of 9,536 hematoxylin- and eosin-stained fragments extracted from 292 full-slide images. The dataset was explicitly designed to facilitate training deep neural networks for classifying colorectal polyps and grading adenomas. Each full slide image in UniToPatho corresponds to a unique patient and has been meticulously annotated by expert pathologists. Annotations divide parts into six distinct classes: NORM (Normal tissue), HP (Hyperplastic Polyp), and TA.HG (Tubular Adenoma, High-Grade dysplasia), TA.LG (Tubular Adenoma, Low-Grade dysplasia), TVA.HG (TubuloVillous Adenoma, High-Grade dysplasia) and TVA.LG (TubuloVillous Adenoma, Low-Grade dysplasia). The study highlights the limitations of using a single deep neural network for accurate tissue type classification. It is stated that a direct classification on a fixed scale is sub-optimal, emphasizing that the distinctive features of each class are extracted at different resolutions. Therefore, the authors propose a new multi-resolution deep learning strategy that includes a set of classifiers. This approach provides a significant improvement and achieves an accuracy of 67%. From a clinical perspective, the most notable results of the study are in the ability to distinguish between tubular and tubulovillous adenomas and accurately grade dysplasia, a particularly challenging task for pathologists. These findings demonstrate the potential of automated approaches to assist with these complex diagnostic tasks. The other dataset in Table 2, CoCaHis^27^ is a database resulting from histopathological images annotated at the pixel level for intraoperative diagnoses. Along with blot-normalized datasets, several machine learning and deep learning classifiers are covered in this work to present their performance across different scenarios. The results have shown the excellence of deep learning classifiers, enabling improvements in intraoperative diagnostic systems. Automatic separation and classification of nuclei in histology images are essential for digital pathology studies, though differences and groupings between nuclei complicate the process. To address these challenges, an artificial neural network called HoVer-Net^28^ was developed (Table 2). It accurately separates and classifies nuclei by measuring the distances of pixels inside the kernels to the kernel center. HoVer-Net consists of three segments: the core-background separation part, the pixel distance calculation part, and the section that determines each core type. The system achieved excellent results across various textures and staining conditions, showing promise for practical applications. HoVer-Net’s ability to discover internuclear spatial relationships and analyze core shapes may provide further diagnostic and prognostic value. HoVer-Net is trained on a single tissue type but is expected to perform well on different tissue types. Future work will focus on improving class balance and exploring the relationship between kernel types and spatial analysis using segmentation masks provided by HoVer-Net. As a result, HoVer-Net offers a powerful solution for the simultaneous separation and classification of nuclei in histology images. The other dataset in Table 2, CRC - TP^29^ proposes a new approach for tissue phenotyping, i.e., the determination of cell types and structures in tissue in colon cancer whole-slide images (WSI). This approach creates cell-cell networks based on potential connections between spatially close cells. The authors use community detection algorithms to identify specific cell groups in complex network analysis. The article recommends combining deep neural networks with community detection algorithms to detect and classify cells. This approach provides a better understanding and analysis of cell groups in tissue and their effect on the overall structure and function of the tissue. In the last dataset in Table 2, NCT-CRC-HE-100K^30^, the researchers aimed to determine whether colorectal cancer (CRC) patients could extract prognostic information from hematoxylin-eosin (HE)-stained tissue slides with a deep learning-based model, convolutional neural network (CNN). Single tissue regions were manually identified on 86 CRC tissue slides, and these regions were used to train CNN with transfer learning. Trained CNN classified nine HE-image classes in an independent dataset of 7,180 images from 25 CRC patients with over 94% accuracy. Using this trained CNN, automated tissue decomposition of representative multi-tissue HE images from 862 slides of 500 stage I-IV CRC patients was performed. A deep stroma score was calculated based on the activations of the CNN output neurons. This score was an independent prognostic factor in a multivariable Cox proportional hazard model for overall survival (OS). The application of computer-aided diagnostics (CAD) systems has received considerable attention in recent years due to their capacity to contribute to the high accuracy and efficiency of medical diagnoses. These result systems have tremendous potential to contribute to the pathological diagnosis in medical imaging of colon polyps, which is very important in the prevention and treatment of colorectal cancer.

The presented study provides a comprehensive dataset of colon polyp and cancer biopsies accumulated from the Kayseri City Hospital Gastroenterology Department in Turkey. It consists of colonoscopy and microscopy images from 201 patients. The dataset comprises 202 colonoscopy videos, totalling 422 minutes, averaging 2.14 minutes per patient, alongside 1903 microscopy images, all collected between 2019 and 2021. This data set makes pathological diagnosis results for 399 polyps available together with the results of immunohistochemical staining for various markers like p53 (clone: bp53-11), Ki-67 (clone: 30-9), CD34 (clone: QBend/10), PD-L1 (clone: SP142), BRAF (clone: V600E), and VEGF (clone: SP125), supplying critical insights into the presence and proliferation of malignant cells. Due to its size, the dataset is primarily suited for benchmarking, model validation, and transfer learning rather than standalone training of deep neural networks. Its open availability will accelerate data-driven advances in detecting, characterizing, and diagnosing colon polyps and cancer. It includes colonoscopy videos, histopathology images, IHC results, and corresponding pathological diagnoses of colon polyps and cancer biopsies. It is comprehensive and multimodal, covering samples from 201 patients with diverse polyp types and cancer stages. This carefully compiled and validated dataset has the potential to provide important contributions to clinicians in their diagnosis and treatment processes while encouraging innovative research on colon polyps and cancer. The description of the dataset suggests that it can be employed in investigating the fundamental colonic polyps and cancer mechanisms and offers a more accurate and efficient diagnosis. This research is very special because high-resolution images and pathological diagnoses for comprehensive data were available, adding to the immense variation in this dataset.

By linking colonoscopy video, matched histopathology slides and IHC profiles from the same lesions, the dataset provides a multimodal resource that is still scarce in colorectal research. Several clinical applications of the dataset, highlighting its distinctive features and pointing out the direction of further research, are presented below:

### Clinical applications

The combined imaging and molecular information can support exploratory studies on diagnosing diverse colonic lesions. The comprehensive data allows one to come up with personalized treatment plans for each patient. This optimizes the treatment choice; hence, it brings forth effective and efficient patient outcomes. Advanced imaging, in concert with molecular analysis, contributes to advances in the early detection of polyps and tumours, especially among high-risk patients. This way, interventions could be offered much earlier, with better clinical outcomes.

### Potential areas for future research

This will be a significant source that could allow comparison with other available data and serve as a benchmark for future studies. Combining genetics and imaging can thus identify new insights into cancer biology and treatment pathways. Associations between IHC data and imaging may provide unique opportunities for understanding the molecular mechanisms of the disease. The nature of the dataset allows for long-term follow-up and prognostic assessment: diseases can be followed up for changes, and the treatment given can be assessed for its effectiveness, which may provide important information regarding prognosis in polyps and cancers. Such studies could ultimately inform more accurate and efficient clinical workflows.

## Methods

### Study population and inclusion criteria

**Fig. 1:**
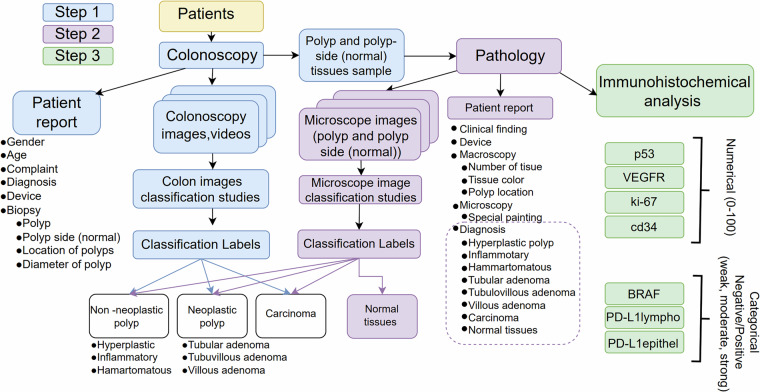
Flowchart of data collection and study.

The data collection process and classification studies for our study are summarized in Fig. 1. This flowchart illustrates the step-by-step process of creating a multimodal dataset by processing patient data through colon imaging (colonoscopy), microscopic examination, and immunohistochemical analysis. The labels derived from the classification studies conducted on the acquired images are also indicated in the diagram.

This case study was conducted on 201 patients, 119 males and 82 females, treated at the Gastroenterology Clinic of Kayseri City Hospital, KCH. In this study, patients with colon polyps were followed from 2019 through 2021 and were followed prospectively for their outcomes. The diagnosis of patients with a colon polyp was by colonoscopy as it is considered the gold standard and a routine procedure. In this colonoscopy examination, the gastroenterologist identified the polyps visually. The study was carried out according to ethical principles and rules of research on human subjects.

Informed consent for participation and data sharing was obtained from all participants in this study. The consent process was applied during colonoscopy and biopsy procedures at the Kayseri City Hospital Gastroenterology Clinic. The study was approved by the KCH Medical Specialization Education Board and the Erciyes University Clinical Research Ethics Committee with decision number 2019/712. Approval was obtained regarding the purpose of the study, data sharing, and use of the results. The inclusion criteria were highly strategic to ensure that the study population was heterogeneous for critical factors such as age, gender, and clinical presentation. This study aimed to collect reliable data from a certain number of patients with a confirmed diagnosis of colon polyps by conducting comprehensive analyses and evaluations. Patients with a genetic family history of colorectal diseases or other conditions that were thought to conflict with the aims of the study were also excluded from the study.

Participants to be included in the study were meticulously selected to ensure no conflicts with patient safety and ethical principles. This approach aims to ensure the validity of the findings and their generalizability to a wider population; thus, increasing the reliability of the study and strengthening its potential in clinical applications. The study included 201 patients, with an average age of 62.74 (±12.39) years. The age distribution among the examined patients ranged from 18 to 91 years, reflecting a wide variability within the study population. Although these findings show that the patient population generally consists of older age groups, the demographic insights obtained should be taken into consideration in subsequent analyses and model generalizability assessments.

The Shapiro-Wilk test, when applied to age data, evaluates whether the data distribution is normal. The test result shows that the age data does not follow a normal distribution since the p-value is less than 0.05. This means the p-value is calculated as 0.0137, smaller than the generally accepted significance threshold of 0.05. This indicates that the age distribution of colon cancer patients deviates from the idealized normal distribution model used to understand clinical and demographic characteristics. These results underline the diverse age profile of patients, emphasizing the importance of accounting for this heterogeneity in understanding patient demographics and disease progression.

**Fig. 2:**
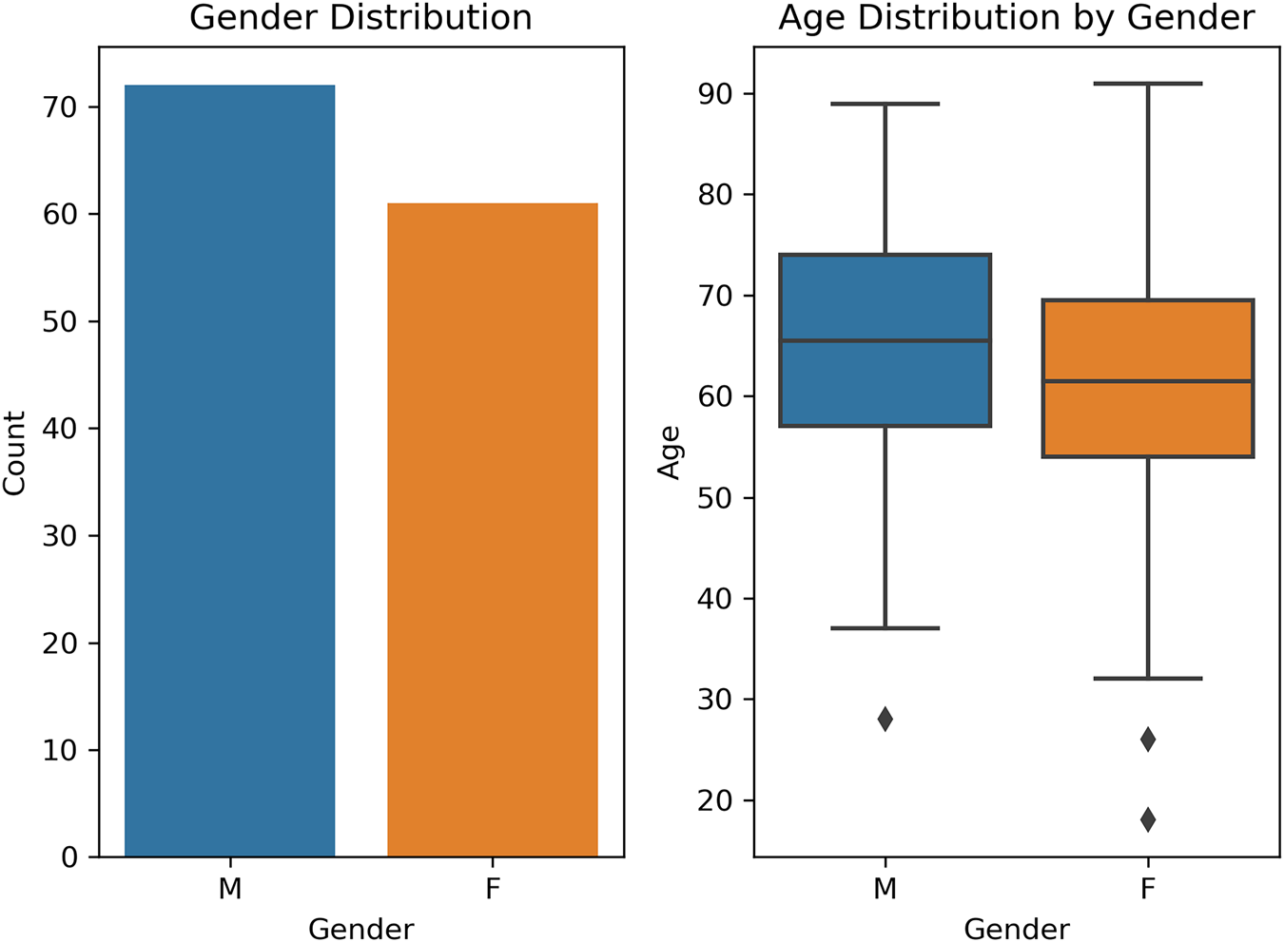
(**a**) Gender distribution of patients (**b**) age distribution by gender.

Figure 2a shows the total numerical distribution of male (M) and female (F) genders. According to the data, there are more male patients than female patients. There are approximately 120 cases in males and approximately 90 cases in females. This graph clearly shows the distribution of colon polyp cases by gender and reveals that male patients are more represented in this study. The age distribution by gender is depicted in Fig. 2b. Differences in age distribution by gender can be observed. The central horizontal line in the boxplot represents the median age. The median age of women is slightly lower than that of men. The upper and lower limits of the boxplot show the distribution of age values in the dataset. Although there is no significant difference in the overall distribution of age values between women and men, the mean age of women is still slightly lower than the mean age of men.

Data integration was achieved using the same patient ID numbers, thus ensuring sustainable analysis of comprehensive and consistent patient-based information. This process allowed multiple types of data, including colonoscopy videos, histopathology images, and IHC results, to be identified and combined using the same patient ID number. This approach facilitated the collection and analysis of polyp data from a single source, increasing data consistency and enabling more robust analyses. Aggregating data using a consistent identification system ensured data integrity and enabled comprehensive patient assessments, contributing significantly to the validity and reliability of the study. By combining multimodal data from the same patient, a more precise analysis of polyp characteristics and a stronger basis for conclusions were created. In addition, this method reduced the issue of missing data, resulting in more consistent analysis results. The meticulous data collection and combination process clarified the study methodology, increasing its transparency and accountability. This further underscores the validity of the study by providing detailed information on how the data were integrated and the reliability of the analyses performed.

### Colonoscopy video and image acquisition

All videos and images taken in this study were obtained from the Kayseri City Hospital Gastroenterology Clinic with advanced HD colonoscopy. The system used in this research was a Fujinon VP-3500HD Processor combined with an XL-4450 light source and a 300 W Xenon lamp. This HD colonoscope provided high-quality, detailed images to support research and add to the current literature. The videos were recorded at a resolution of 1920 × 1080 pixels to provide high-resolution video recording for in-depth investigation and analysis. The colonoscopy procedures included in this dataset were performed by an experienced gastroenterologist by directly examining the inner surface of the colon through a flexible, lighted tube (colonoscope) inserted through the rectum. The entire process was recorded using a High Definition (HD) endoscopic device.

Imaging aside, tissue samples were obtained from the polyps’ sites using various equipment, such as forceps or snares, and prepared for histopathology. For a comparative study, samples of normal colonic mucosa next to the polyp are also taken and analysed. This allows for a comprehensive examination of both polyp and normal tissue. These samples can be taken from the areas surrounding the polyp during the colonoscopy procedure. Collecting samples of both polyp and normal tissue will allow for detailed examination and comparison of both types of tissue. Although these normal tissues are not available for every polyp, further details can be obtained from the histopathological image dataset section and are indicated on the labels of the images. All video recordings and photographs of colonoscopy will be stored securely for further evaluation and documentation. Patient data confidentiality and privacy have been secured with appropriate respect to applicable ethical and statutory laws.

This provided sufficient ground for further analysis and research to gain a thorough knowledge of colon polyps and their pathological consequences.

In our study, the total duration of 202 colonoscopy videos was initially 422 minutes, and the average duration of each video was determined as 2.14 minutes. However, these average values accurately reflect the actual duration of the video belonging to the patient; the relevant standard deviation was calculated as ±4.35 minutes. A rigorous video selection process was applied to ensure one-to-one label matching for supervised learning. In this process, 35 videos were excluded because they did not meet our quality standards or were not informative enough. In addition, 34 videos containing more than one polyp were not included in the analysis because the problem with a specific polyp in the video could not be reliably provided. The results of these eliminations were classified as 133 colonoscopy videos that were correctly labelled according to the pathological diagnosis of colon polyps. The total duration of these selected videos was 168 minutes, and the average duration of each video was set as 1.29 minutes. The standard deviation of these durations was determined as ±2.35 minutes, and it was shown that these video durations could deviate from the mean by ±2.35 minutes. Due to the requirement of one-to-one label matching, only these 133 videos can be used for supervised learning activities; this is an important factor to consider in the temperature limitation of the data setting and when designing machine experiments in video-based training tasks.

However, in this case, it does not affect other data modalities, including IHC studies. This video selection is valid for researchers who want to perform polyp analysis from recorded colonoscopy videos only. For histopathology analyses, the dataset is collected from 1903 microscopy images obtained from 383 different polyps belonging to 198 patients, and summarized pathological labels are provided for these samples regardless of the availability of the corresponding video. Similarly, researchers working independently of the videos can use the borders of the problems and IHC points directly or the microscope images together with IHC parts.

**Fig. 3:**
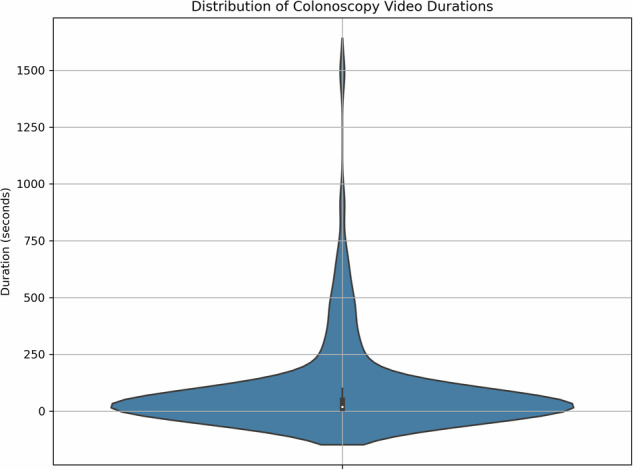
Violin plot of colonoscopy video durations (seconds); x-axis truncated at 600 s for readability.

The plot illustrates in Fig. 3 the density and spreads of durations across all videos. A right-skewed distribution is observed due to a few very long videos. Figure 3 show that the distribution of colonoscopy video durations is right-skewed, with most videos concentrated below 100 seconds. Many outlier videos extend beyond 500 seconds, which may bias the distribution. To better visualize the general pattern, the longest video was excluded in some plots. These visualizations provide a clearer understanding of the dataset structure and ensure appropriate modelling and preprocessing decisions.

However, an Excel file also provided information about the patient and the video. This dataset includes demographic information, pathology results, and colonoscopy videos, facilitating the use and analysis of video data while protecting patient privacy. The dedicated work and data collection procedure could establish a new standard in the study of colonoscopy videos and serve as a valuable resource for future research.

**Fig. 4:**
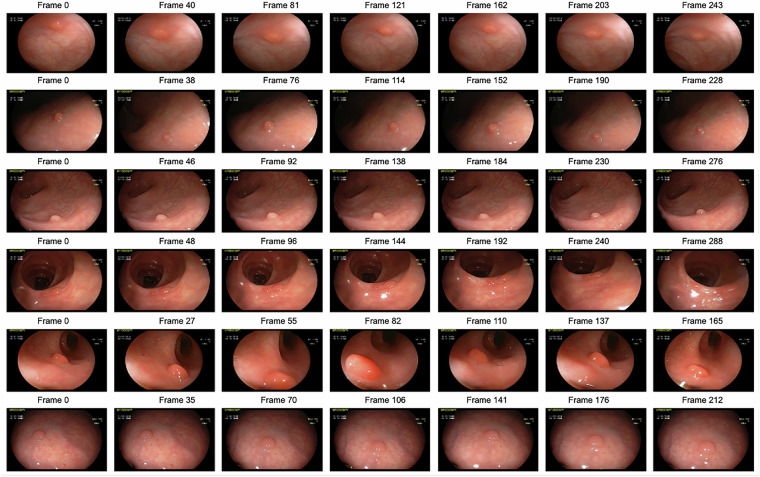
Examples of extracted frames from videos.

Figure 4 illustrates some examples of extracted frames from colonoscopy videos of different patients in our dataset. These frames give an idea of essential features such as polyp variety, size, morphology, and colour.

Each video was taken during a colonoscopy examination and provides a variety of images of colon polyps. These visual examples demonstrate that our dataset offers a comprehensive and diverse collection of colon polyp images. A simple non-informative-frame filter based on colour variance, previously validated on our endoscopy footage^31^, can be applied as a first preprocessing step. Initial experiments on vessel-density based localisation in colonoscopy images achieved ≥0.82 F1-score for frame-level polyp detection^32^, underscoring the value of handcrafted vascular cues that can be revisited with the present dataset.

**Fig. 5:**
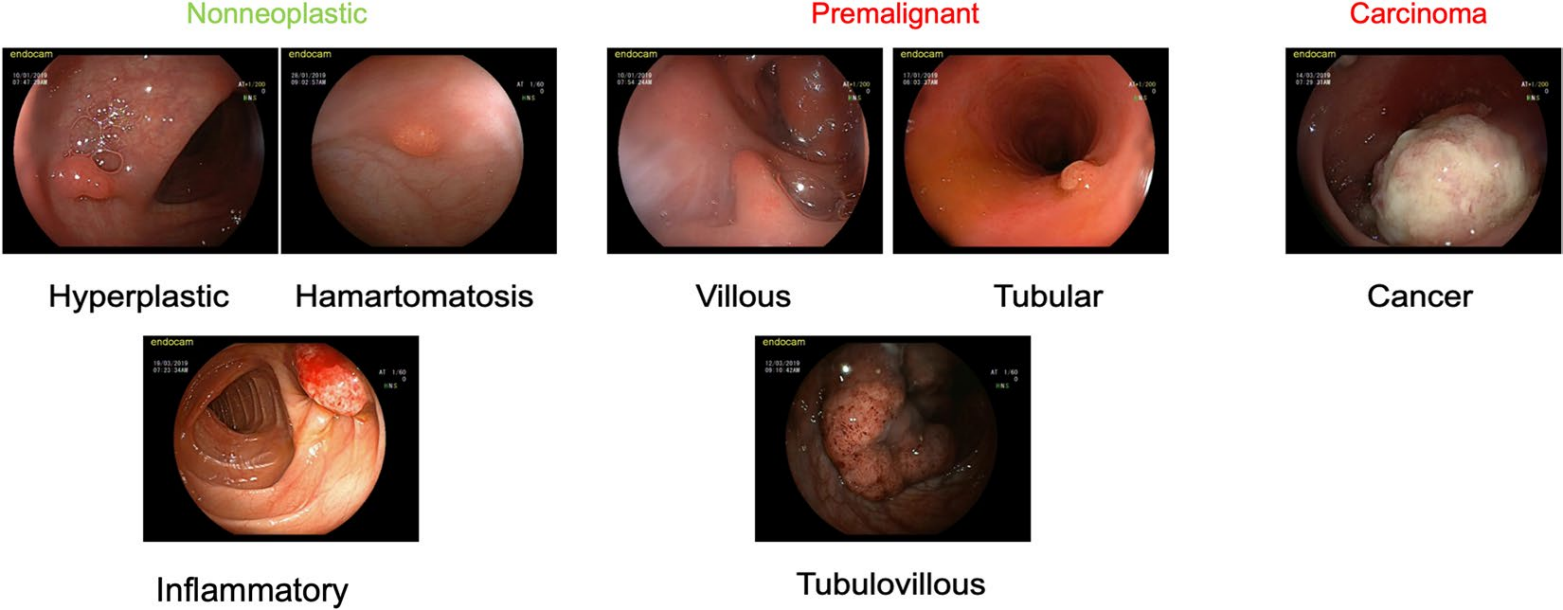
Examples of the different polyp types.

High-quality and high-resolution images are essential in increasing the accuracy and reliability of automated polyp detection and classification systems. These annotated images (Fig. 5) allow researchers and clinics to understand better the appearance of colon polyps and the nature of the images for training and evaluating automated polyp detection and classification systems.

**Fig. 6:**
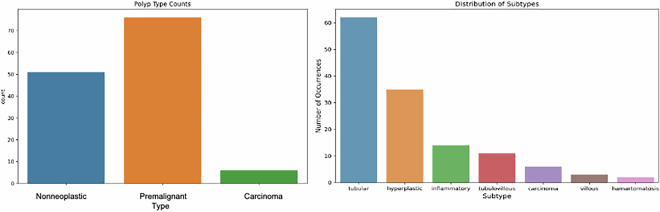
The distribution of polyp types and subtypes colon video input dataset.

Figure 6 indicates the distribution of polyps in the data set according to their types and subtypes in the classification/characterization studies to be done using column videos. There are 133 colon polyps in total. Of these polyps, 76 were identified as premalignant, 51 as nonneoplastic, and six as carcinomas. This video set represents the diversity of colonic polyp types, with tubular polyps being the most defined and hamartomatosis and villous polyps being the least described. Sessile serrated polyp (SSP) cases were also observed but, due to their very limited number, they were merged under the hyperplastic category in this release. This distribution provides essential information about how the dataset can be used to train classification models. Class imbalance in the dataset can affect the model’s ability to predict different classes and should be considered. As a result, this database of colonoscopy videos provides a valuable and comprehensive resource in colon polyp classification research.

In this study, no class is defined as normal in studies using colonoscopy videos because tissue samples labeled as normal from colonoscopy videos could not be detected accurately. Only microscope images and immunohistochemistry (IHC) results were used to determine the presence of normal tissues in the analyses. If it is desired to analyze normal tissues in the study, datasets consisting of microscope images and IHC results should be preferred. Because colonoscopy videos do not have labels for normal tissues, it is necessary to consult other datasets to analyze normal tissues. This approach makes obtaining more accurate and reliable results in an analysis of normal tissues possible.

### Histopathological image acquisition

Histopathological image acquisition was an important step since it was part of characterizing further and analyzing the colon polyps detected by colonoscopy. Also, biopsy samples from the polyp and the normal colonic mucosa around it were sent to the Pathology Clinic at Kayseri City Hospital for processing and subsequent histopathological investigation. Upon arrival at the Pathology Clinic, biopsy samples were received and fixed by following the standard process. To maintain tissue morphology and the prevention of degradation, the samples were fixed in the 10 percent formalin solution. Then, the samples were embedded in paraffin blocks to enable good sectioning of samples. For the preparation of histopathological specimens, paraffin blocks carrying specimens were cut into sections 5 µm thick with the help of a microtome. These tissue sections were stained by H&E, one of the most used staining techniques in histopathology, helping to bring out the cellular structure and organizational patterns within the tissues. H&E staining allowed the pathologists to investigate colon tissue samples for cellular architecture, inflammatory responses, and other pathological features. The slides prepared were observed in a light microscope Nikon Eclipse NI with lenses that provided different magnifications. The tissue morphology, cellular abnormalities, dysplasia, and characteristic features of the polyp were evaluated by a qualified expert pathologist assessment of histopathology. High-resolution images were captured using a Nikon DS-Fi2 camera installed on the microscope, recording histopathological findings. These photos depicted the cellular structures, dysplastic changes, and other histological characteristics associated with colon polyps.

Other than the histopathological examination, an IHC was done to explore the expression of some proteins in polyp tissues. The subsequent detailed description of the IHC procedure, including the choice of antibodies and staining, will extend the explanation of current IHC methods.

There are 1903 high-resolution whole-slide histopathology images of 198 patients and 383 distinct polyps. The images were captured by a slide scanner using different magnifications, such as 20X and 40x. Patient data includes demographic information such as age and gender and the pathology results related to protecting patient confidentiality. Two experienced pathologists performed pathological diagnoses. The image labelling for each polyp is assigned a diagnosis, such as hyperplastic polyp, adenomatous polyp, or carcinoma.

**Fig. 7:**
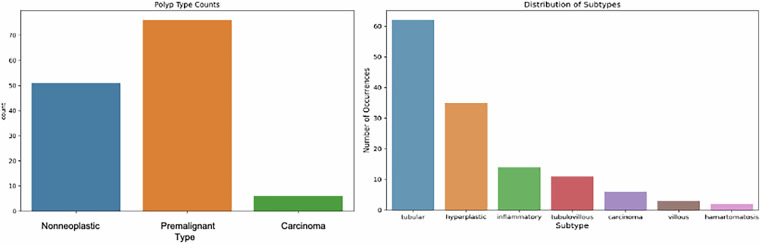
The distribution of polyp types and subtypes of histopathology images input dataset.

Our dataset is divided into four categories: nonneoplastic (benign), premalignant, carcinoma, and normal (Fig. 7). These categories represent pathological conditions determined as a result of microscopic examination of patients’ tissues. The largest group is neoplastic, with 940 samples. The nonneoplastic category represented the second largest group, with 519 samples, while the normal and carcinoma categories were less defined, with 370 and 74 samples, respectively. There are also more specific classifications under the premalignant category. These subcategories are carcinoma, hamartomatosis, hyperplastic, inflammatory, normal, tubular, tubulovillous, and villous. When the distribution of these subgroups is examined, it is seen that the largest group is tubular, with 738 samples, while the smallest group is hamartomatosis, with 21 samples. Sessile serrated polyp (SSP) cases were merged under the hyperplastic category due to limited sample size within this dataset release. These class distributions show the diversity of the data set and the detailed classification options. This diversity enables the model to learn a broader range of pathology and thus be used in a more comprehensive range of applications. It is important for us to determine how effective our model is in classifying colon polyps.

We acknowledge the presence of class imbalance across polyp types. While no resampling was applied in this dataset release, we recommend stratified sampling or cost-sensitive training methods when applying ML models to this dataset.

**Fig. 8:**
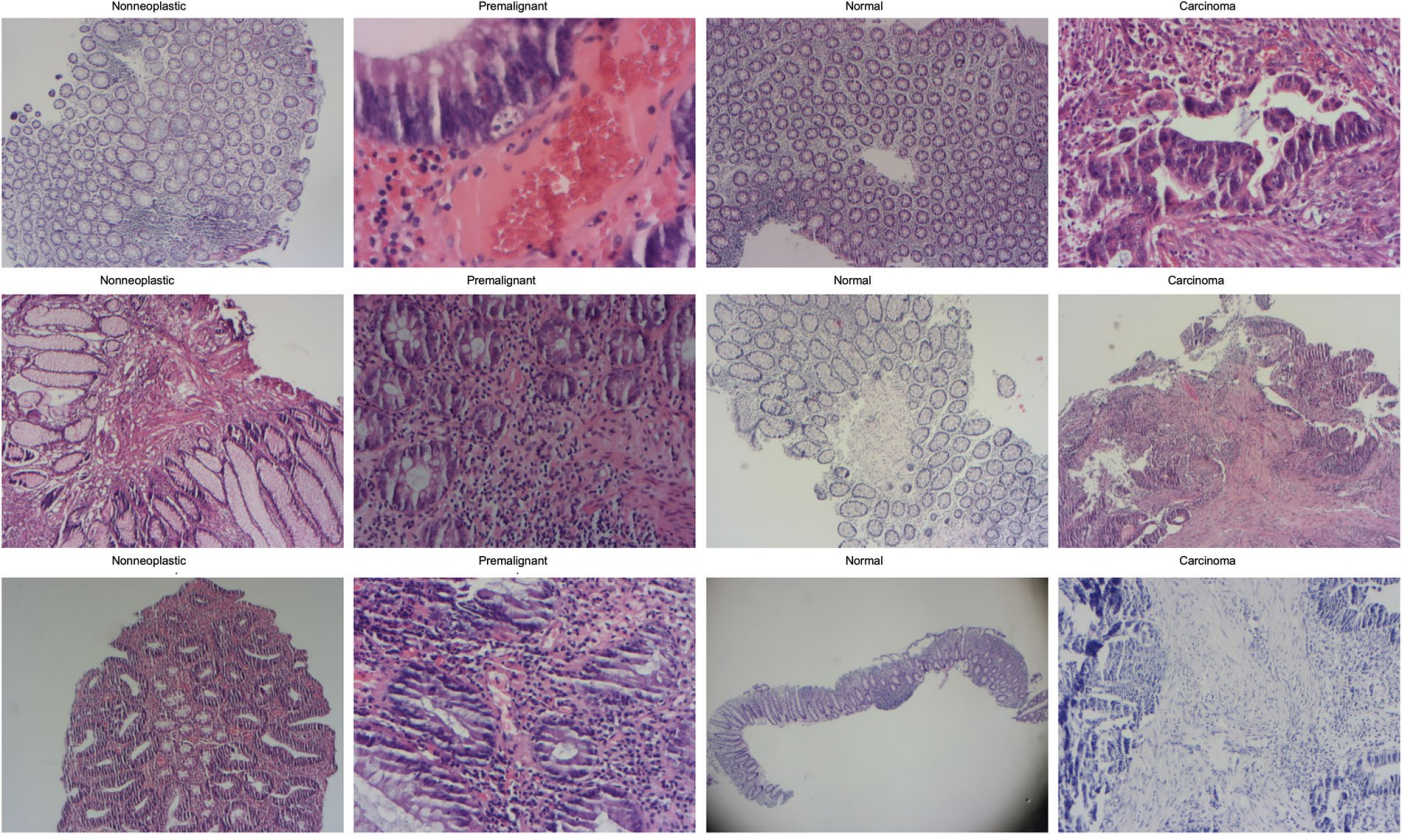
Nonneoplastic, premalignant, normal, and carcinoma examples from the input image set.

In Fig. 8, nonneoplastic, premalignant, normal, and carcinoma samples are shown with images of various histopathological sections of colon tissues. These images provide the opportunity to visually compare disease states and tissue structural differences. Nonneoplastic polyps refer to benign entities (hyperplastic, inflammatory, hamartomatous). Precancerous lesions are reported under the premalignant category (tubular, tubulovillous, villous). Additionally, the normal tissues represent healthy colon mucosa, unlike carcinoma samples, which represent malignant tumours, i.e., cancerous tissues. These images provide important research and clinical diagnosis information by clearly showing how different tissue types appear under the microscope.

**Fig. 9:**
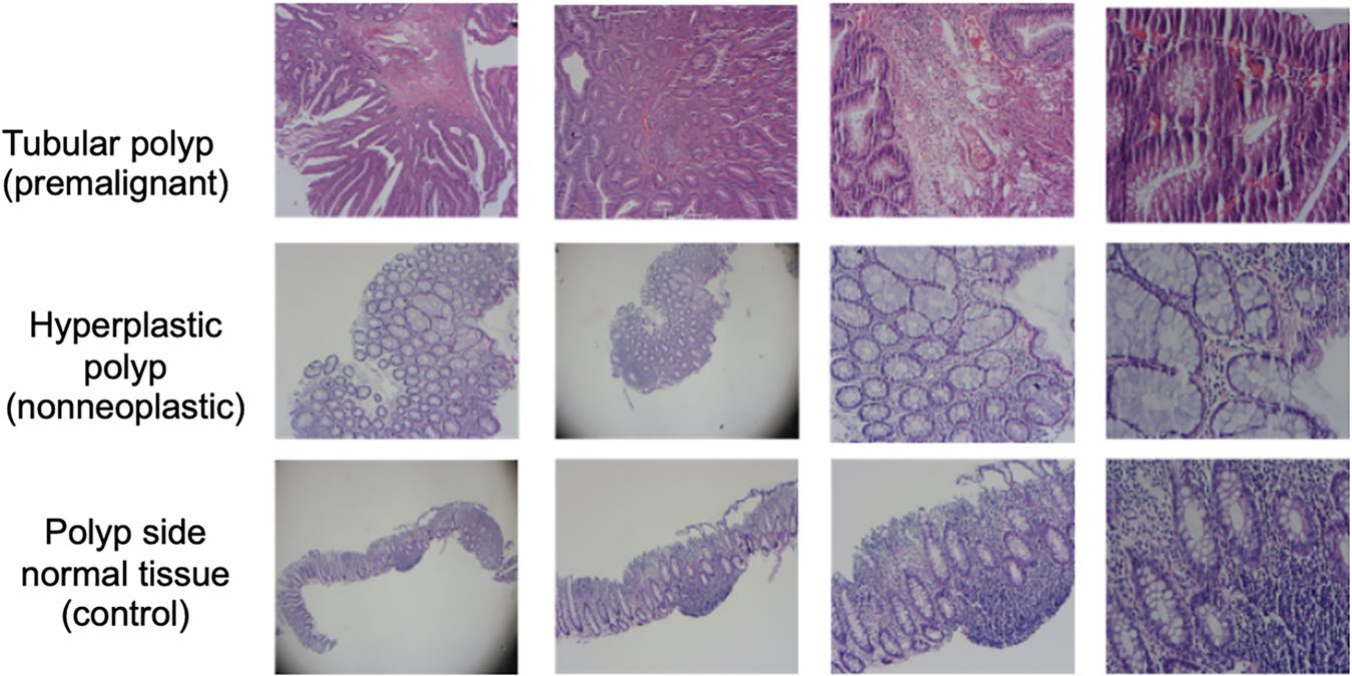
Different polyp types from the same patient with all magnification levels (2.5X, 5X, 10X, 20X from left to right).

Within the scope of this study, microscopic images were obtained in a total of five different subfolders at different zoom levels. The folders are named ‘2.5X’, ‘5X’, ‘10X’, ‘20X’ respectively in Fig. 9. The number of images in each subfolder is as follows: 2.5X folder contains 393 images, 5X folder contains 397 images, 10X folder contains 400 images, 20X folder contains 399 images, and 40X folder contains 316 images.

**Fig. 10:**
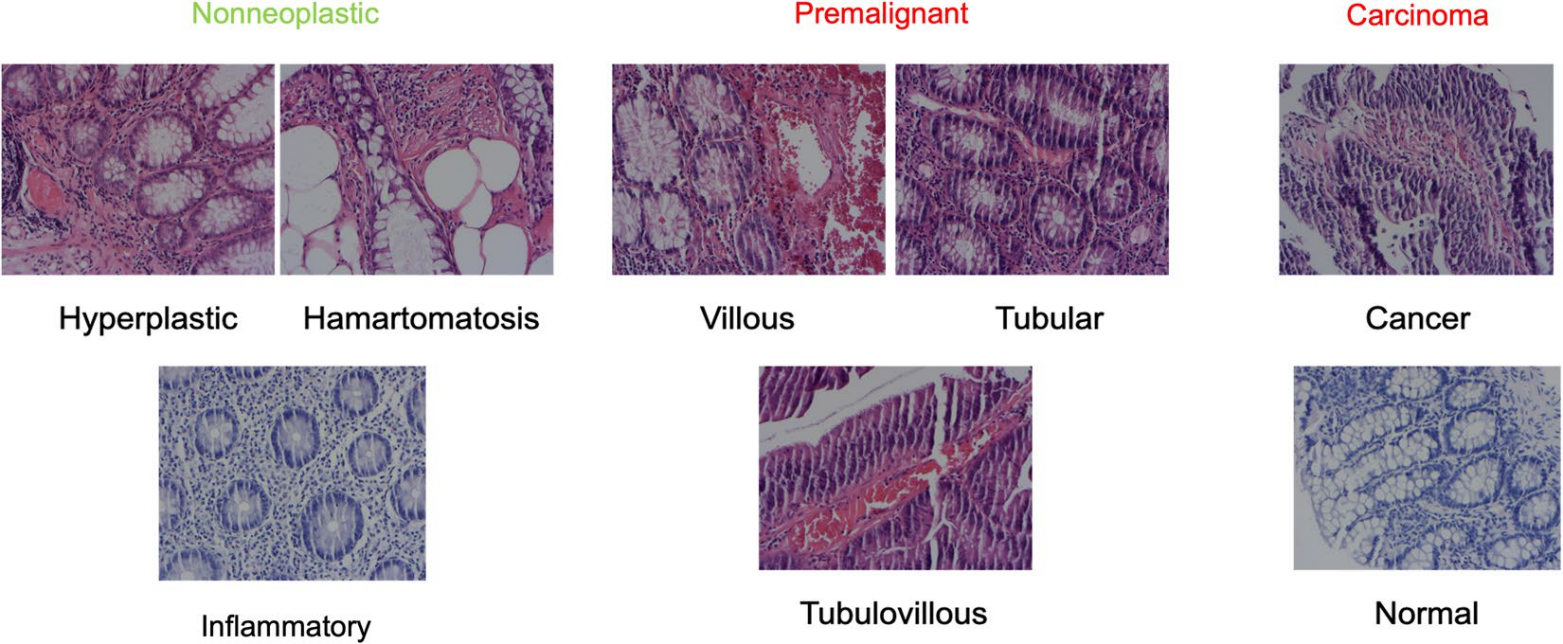
An example image from all types.

Figure 10 shows the diversity of the various tissues in the dataset, which are examined under the microscope at different zoom levels; this diversity provides a wide field of view for pathological analysis and diagnosis. The dataset comprises normal and pathological tissues, making it a valuable resource for training and evaluating machine learning models.

**Fig. 11:**
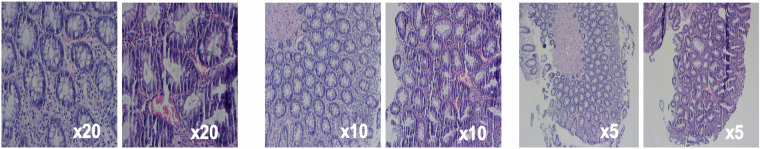
Sample images of tubular adenoma polyp and normal tissue samples taken from the side of the same polyp, respectively.

The ‘2.5X’ view provides the most general view of the tissue, revealing large-scale structures and the overall texture layout. In contrast, the ‘5X’, ‘10X’, and ‘20X’ images provide higher resolution, allowing cells and cellular allows for a more detailed examination of the structures (Fig. 11).

### Immunohistochemical staining and scoring

The dataset was generated using formalin-fixed paraffin-embedded (FFPE) tissue samples from colon polyp cases. Sections were taken from each paraffin block, and immunohistochemical (IHC) staining was performed using the Ventana Benchmark XT system. Specific antibodies were carefully selected to target proteins such as p53 (clone bp53-11)^33,34^, Ki-67 (clone 30-9)^35,36^, CD34 (clone QBend/10)^37,38^, PD-L1 (clone SP142)^39–41^, BRAF (clone V600E)^42–44^, and VEGF (clone SP125)^45^, which play an important role in colon cancer research. These antibodies were selected considering their importance in colon cancer studies. The staining procedure was performed by standard cell conditioning protocols, including steps that enhance antibody-antigen interactions, such as deparaffinization and antigen retrieval. Tissue sections were incubated with the selected primary antibodies to ensure specific binding to target proteins. Antigen-antibody complexes were visualized using secondary antibodies and chromogen detection techniques, thus obtaining a comprehensive data set containing protein expression patterns and tissue structures.

Table 3 shows a representative example from the IHC dataset. The dataset provides quantitative values for markers such as Ki-67, VEGF, CD34, and p53, expressed on a continuous 0–100 scale, and categorical scores for markers such as BRAF and PD-L1 (negative, weak, moderate, strong).

**Table 3:** An example from the IHC dataset.

Ki-67 (clone 30-9)	BRAF (clone V600E)	PD-L1epithel (clone SP142)	PD-L1lympho (clone SP142)	VEGF (clone SP125)	CD34 (clone QBend/10)	CD34 (clone QBend/10) score	p53 (clone bp53-11)
50	negative	negative	negative	20	47	2	15
40	negative	negative	negative	30	39	2	10
50	negative	negative	negative	50	50	3	20
50	negative	negative	weak	40	63	3	5
60	negative	negative	weak	70	57	2	25
10	negative	negative	negative	40	70	3	2
30	negative	negative	weak	75	50	2	25
15	negative	negative	negative	0	55	2	5
10	negative	negative	negative	0	34	1	5
5	negative	negative	weak	20	45	2	3

**Fig. 12:**
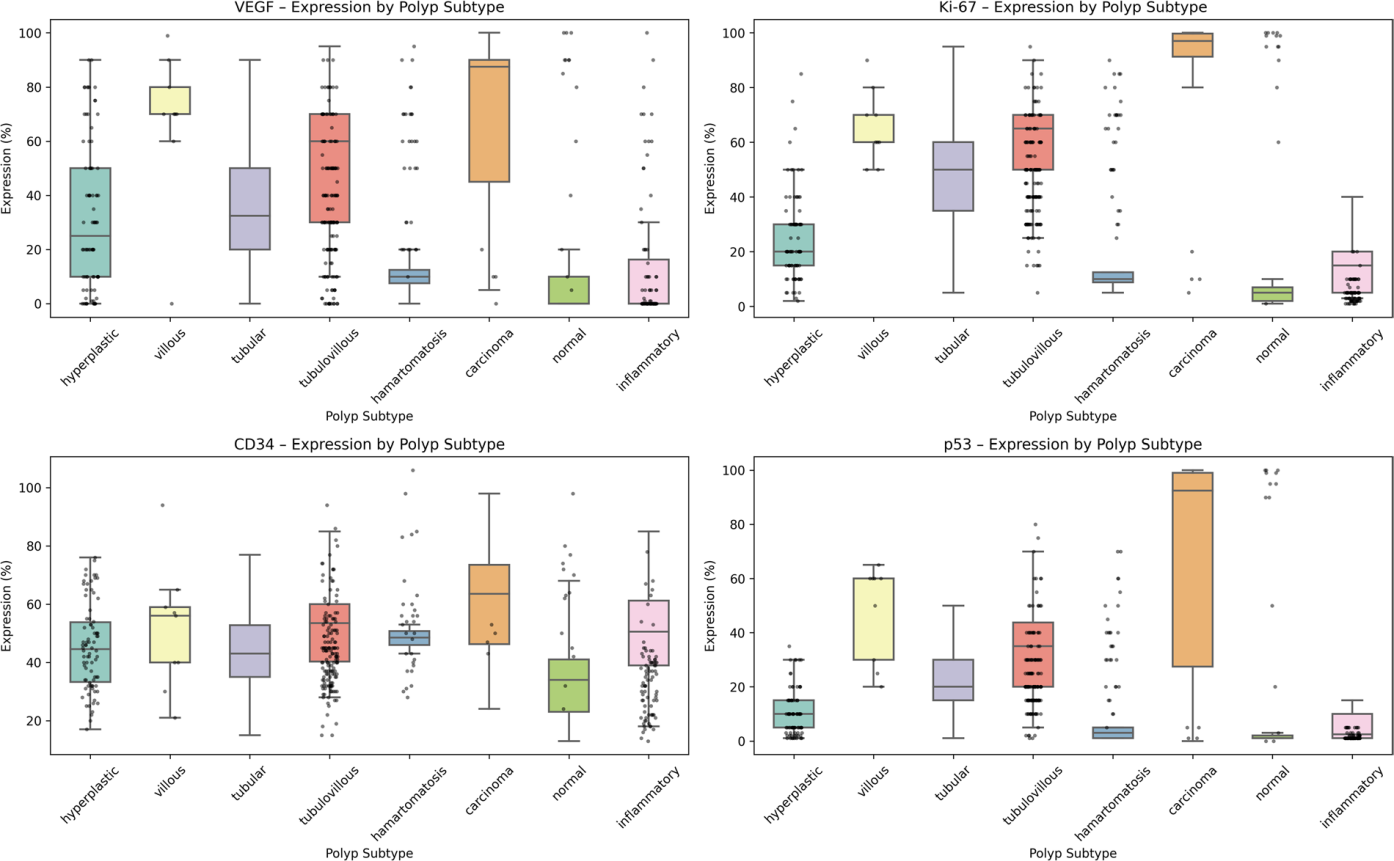
Box and strip plots showing the expression levels of four prognostic IHC markers (VEGF, Ki-67, CD34, and p53) across different polyp subtypes.

Figure 12 presents box and strip plots of VEGF, Ki-67, CD34, and p53 expression levels across various polyp subtypes. Each boxplot indicates the median, interquartile range, and outliers, with overlaid dots representing individual polyp-level measurements. These plots summarize the available marker distributions for different subtypes in the dataset.

**Fig. 13:**
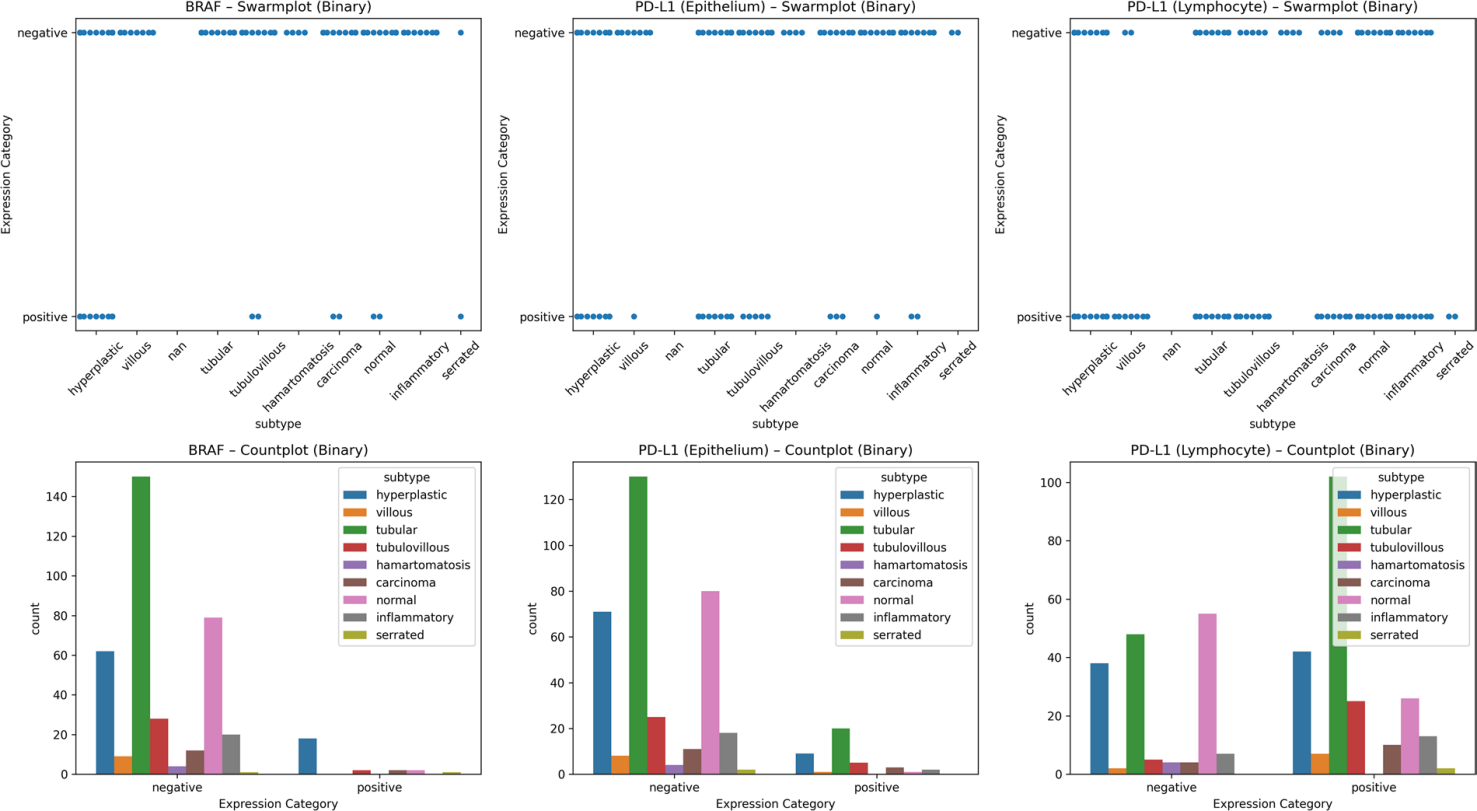
IHC expression patterns by polyp subtype.

Figure 13 shows the distribution of BRAF and PD-L1 expression patterns across colon polyp subtypes. The top row presents swarm plots for expression categories (negative, weak, moderate, strong), while the bottom row shows count plots illustrating category frequencies by subtype. These visualizations provide an overview of categorical distributions of immune-related markers across different subtypes.

Table 4 illustrates how demographic information (Step 1), histopathological classification (Step 2), and immunohistochemical (IHC) marker scores (Step 3) are linked at the polyp level. Each row represents a unique polyp identified by patient and polyp IDs. This integrated structure allows researchers to cross-reference clinical, pathological, and molecular features for comprehensive multimodal analyses.

**Table 4:** Example of integrated dataset.

Step 1						Step 2	Step 3							
Demographics						Pathologics	Immunohistochemical (IHC) markers							
ID	patient	polyp	gender	age	location	subtype	Ki-67	BRAF	PD-L1epit	PD-L1lym	VEGF	CD34	CD34 score	p53
1	patient1	polyp1	M	53	descending	hyperplastic	50	negative	negative	negative	20	47	2	15
1	patient1	polyp2	M	53	descending	hyperplastic	40	negative	negative	negative	30	39	2	10
1	patient1	polyp3	M	53	descending	hyperplastic	50	negative	negative	negative	50	50	3	20
2	patient2	polyp1	M	49	ascending	hyperplastic	50	negative	negative	weak	40	63	3	5
2	patient2	polyp2	M	49	descending	villous	60	negative	negative	weak	70	57	2	25
3	patient3	polyp1	M	70	ascending	hyperplastic	10	negative	negative	negative	40	70	3	2
4	patient4	polyp1	F	57	sigmoid	hyperplastic	30	negative	negative	weak	75	50	2	25
5	patient5	polyp1	F	65	ascending	hyperplastic	15	negative	negative	negative	0	55	2	5
5	patient5	polyp2	F	65	ascending	hyperplastic	10	negative	negative	negative	0	34	1	5
5	patient5	polyp3	F	65	descending	hyperplastic	5	negative	negative	weak	20	45	2	3

## Data Records

The dataset is structured into three primary modalities: colonoscopy videos, histopathology images, and immunohistochemistry (IHC) results, forming a multimodal for the study of colorectal polyps. Each component is stored in a separate directory and can be cross-referenced using patient and polyp identifiers encoded in the file names.

### Colonoscopy video data set

The dataset’ colonVideosWithLabels’ contains 133 colonoscopy video files in.avi format. Each video corresponds to a single polyp and includes structured metadata encoded in the file name, such as Patient ID, sex and age, polyp location (e.g., sigmoid, ascending), polyp type (e.g., nonneoplastic, hyperplastic), IHC results for selected markers (e.g., p53, Ki-67, PD-L1, VEGF, CD34).

Additionally, an Excel file provides a structured index to all videos and their corresponding labels.

Example filename:

Directory Architecture:

-colonVideosWithLabels

-colon-patient3-polyp1-M-70-ascending-nonneoplastic-hyperplastic-10-negative-negative-negative-40-3-70-2.avi

-colon-patient4-polyp1-F-57-sigmoid-nonneoplastic-hyperplastic-30-negative-negative-weak-75-2-50-25.avi

### Histopathological image data set

The dataset’ pathoImagesWithLabels’ includes high-resolution histopathological images of polyps in TIFF format. Each polyp is represented across multiple magnification levels (2.5X, 5X, 10X, 20X, 40X). The file names encode patient and polyp ID, polyp classification, tissue location (e.g., descending colon), slide number and magnification. Images are grouped into subfolders by magnification level. An accompanying Excel file, ‘pathoImagesWithLabels.xlsx,’ includes detailed IHC scores corresponding to each histopathological image.

Directory Architecture:

- pathoImagesWithLabels:

- Magnification Level 1: 2.5X

- patho-patient1-polyp2-descending-nonneoplastic-hyperplastic-slideX2,5.tiff

- Magnification Level 2: 5X

- patho-patient1-polyp2-descending-nonneoplastic-hyperplastic-slideX5.tiff

- Magnification Level 3: 10X

- patho-patient1-polyp2-descending-nonneoplastic-hyperplastic-slideX10.tiff

### Immunohistochemistry (IHC) results data set

The last dataset, ‘ihc_data.xlsx,’ provides detailed protein expression scores for each polyp based on immunohistochemical staining. Expression levels are provided as either categorical (e.g., “positive”, “negative”, or “weak”) or percentage scores. Markers include: p53, Ki-67, VEGF, PD-L1, CD34, BRAF.

This dataset includes IHC results for tissues that may not have corresponding colonoscopy videos or histopathology images. The IHC data can be used independently for studies focused solely on IHC analysis, but it can also be cross-referenced with the other two datasets using unique patient identification numbers. This cross-referencing allows for a more comprehensive analysis involving colonoscopy videos, histopathological images, and IHC results. In the released dataset, the original labels follow the simpler convention of “neoplastic” and “nonneoplastic”. To avoid confusion and to be consistent with pathology standards, in this manuscript we refined the terminology as follows: Nonneoplastic (benign) polyps: hyperplastic, inflammatory, and hamartomatous, premalignant polyps: tubular, tubulovillous, and villous, carcinoma: listed separately but considered under the neoplastic process, normal tissues: remain consistent across dataset and manuscript.

This means that while the folder names and filenames in the dataset still contain the terms “neoplastic” and “nonneoplastic,” the categories are described in the manuscript using the more precise terminology above. Researchers using the dataset can therefore map “neoplastic” labels in the raw data to “premalignant” (for tubular, tubulovillous, and villous).

The dataset has been deposited in Zenodo and is openly accessible at 10.5281/zenodo.15388073^46^.

## Technical Validation

All images were taken under the same conditions using the same colonoscopy, microscope, and camera settings. Second, we introduced negative controls into our data collection process—negative controls formed by images of correctly labeled normal colon tissue—so that our classification system can describe what sets normal from abnormal tissue.

A robust approach to the classification and identification of subtypes of polyps was used in the present study. We combined the powers of two highly effective staining methodologies: HE and IHC. Double staining is used in the present study because it may improve the accuracy and reliability of the results. The consistency of the results obtained by these two methods was tested using the well-known and widely used statistical measure of Cohen’s Kappa because it can precisely quantify the degree of agreement between two nominal-scale assessments. Our analysis showed a high agreement between the two methods; the Kappa coefficient was 0.9. It will confirm the high consistency and reliability of our diagnostic approach. Our earlier study on large public histopathology sets demonstrated that careful optimisation of network depth and stain-normalisation is critical for reliable polyp grading^47^. Although that work used a different public dataset, the methodology is directly transferable to the slides released here.

As another statistical measure, a percent agreement calculation was also done simultaneously, which aims to depict the degree of agreement between the two different staining techniques: HE and IHC. From this measurement, approximately 90.41% can be obtained. That is, both the HE and IHC staining techniques result in similar results from polyp classification. Therefore, both techniques were highly reliable and compatible within the framework of our investigation. The double-staining strategy yields consistent, unquestioned results, which supports our results, thus further consolidating the statements made in this paper. A high concordance of the HE and IHC techniques gives intrinsic validity to our approach, pointing out the potential for our double-staining strategy in current and future histopathological research and further clinical practice.

Blanking was done so that no prior knowledge would impact the image classification output. Also, all samples were carefully followed up from the collection stage to the analysis stage for proper and reliable identification. The technical validation section herein supports the dependability of the dataset and confirms its fitness for the following levels of analysis and interpretation. In this regard, we avoided the prior knowledge of the expected outcomes to retain the findings validity, leading to more reliable and unbiased interpretations. We consequently contend that this retains the strength of the dataset and ascertains that any future research has something concrete on which to rely.

An important limitation of this study is that the dataset was collected from a single center (Kayseri City Hospital, Turkiye). This may limit the generalizability of the findings to patient populations in different geographic regions or centers with different clinical protocols (e.g., colon preparation methods). Future studies may overcome these limitations by integrating multicenter datasets that are more representative of larger populations and include more comprehensive demographic information. Preliminary experiments on feature-selected models achieved 78% balanced accuracy for hyperplastic vs tubular adenoma discrimination even with limited samples^48^, highlighting the viability of classical ML pipelines on modest-sized subsets. On conventional white-light and NBI colonoscopy images, a histogram of oriented gradients + Random Forest pipeline reached 95% accuracy in binary polyp-type discrimination, outperforming expert endoscopists, whereas a simple CNN was superior in the 3-class setting^49^. These findings motivate hybrid ML/DL baselines on the VIM-Polyp videos^50^

In this respect, there is an urgent need for broad and high-quality colorectal polyp histopathology image datasets to enable advanced research into CAD systems capable of supporting pathologists in diagnosing and classifying colon polyps. Such a dataset would foster innovation in colon polyp research, improving clinical diagnostics and patient outcomes. The incidences of colon polyps and cancers are among the most prevalent gastrointestinal disorders, with increasing rates worldwide. Early detection and diagnosis are crucial for timely treatment and improved patient outcomes. Currently, the gold standard of diagnosis involves colonoscopy and biopsy, followed by pathological examination. These procedures take time and may vary among pathologists. Hence, there is a need for more appropriate diagnostic tools to improve patient care quality.

## Data Availability

The VIM-Polyp dataset has been deposited in Zenodo and is openly accessible at 10.5281/zenodo.15388073.

## Code availability

The code used to generate and process the dataset described in this manuscript is publicly accessible on Github, a well-known platform for data science and machine learning. The repository includes detailed documentation with comments to help researchers navigate and utilize the code. Researchers interested in accessing the code can visit the following link: https://github.com/biorsd/VIM-Polyp. The code is freely available for download and can be explored, replicated, or modified to support further research.

The code was developed using Python version 3.8 and utilizes essential libraries, including:

• **Scikit-learn (version 0.24.2)** for machine learning tasks.

• **Matplotlib (version 3.4.3)** for data visualization.

To ensure reproducibility, the code includes clear variable names and thorough comments detailing the purpose and functionality of each section. It also specifies the variables and parameters for generating, testing, and processing the datasets. Researchers are encouraged to consult the code documentation for a deeper understanding and to modify it to fit their specific research goals.
